# Optimizing axial length estimation for intraocular lens power calculation in phacovitrectomy for macula-off retinal detachment

**DOI:** 10.1186/s40942-025-00666-5

**Published:** 2025-04-02

**Authors:** Sukhum Silpa-archa, Chitchanok Samanwongthai, Variya Nganthavee, Korawin Charoensuk

**Affiliations:** Department of Ophthalmology, Rajavithi Hospital, College of Medicine, Rangsit University, 2, Phayathai Road, Ratchathewi District, Bangkok, Thailand

**Keywords:** Axial length, Phacovitrectomy, Macula-off retinal detachment, Intraocular lens power calculation

## Abstract

**Background:**

To evaluate methods of preoperative axial length (AL) estimation for intraocular lens (IOL) power calculation in patients with macula-off rhegmatogenous retinal detachment (RRD). These methods included optical biometry, A-scan biometry, and novel decision algorithms.

**Methods:**

A retrospective analysis of prospectively collected data was conducted at a tertiary hospital from January 2018 to December 2023. Preoperative and postoperative AL measurements were obtained using optical biometry (IOL Master 700, Zeiss, Germany) and A-scan biometry (VuMAX, Sonomed, USA). The primary outcome was the mean absolute prediction error (MAE) between postoperative AL and preoperative estimates generated by five methods, including two novel algorithms.

**Results:**

The study included 56 patients (56 eyes). The lowest MAE was achieved using the simple algorithm (0.31 ± 0.55 mm), followed by the AL of the fellow eye measured via IOL Master (0.34 ± 0.60 mm), and the advanced algorithm (0.36 ± 0.62 mm). A Kruskal-Wallis H test found no statistically significant difference in MAE across the five methods (*P* = 0.118). Bland-Altman analysis demonstrated good agreement between preoperative and postoperative AL measurements obtained with the IOL Master.

**Conclusion:**

For patients undergoing phacovitrectomy for macula-off RRD, the simple algorithm provides accurate AL estimation for IOL power calculation. In cases where AL measurement of the affected eye is not feasible using the IOL Master, the fellow eye’s AL is a reliable alternative.

## Introduction

Combined phacovitrectomy has become a common procedure for many vitreoretinal diseases, including rhegmatogenous retinal detachment (RRD) for which phacoemulsification is indicated. The advantages of combined phacovitrectomy over vitrectomy with delayed cataract surgery include a stress-free vitreous shaving procedure without concern for intraoperative lens damage, reduced time and cost of surgery, and faster visual recovery [1, 2]. Nevertheless, in patients with retinal detachment requiring phacovitrectomy with intraocular lens (IOL) implantation, the accuracy of IOL power calculation is crucial, as well as challenging to achieve, because a detached macula affects axial length (AL) measurements. Given the pathophysiology of the disease, the AL is shorter and tends to be less accurate in eyes with macula-off RRD, and postoperative refraction tends to be myopic since the IOL power is overestimated [3].

A few studies have evaluated the accuracy of preoperative AL estimation techniques for IOL power calculation for combined phacovitrectomy in macula-off RRD. These have included optical biometry and A-scan for same and fellow eyes [4–7], user-adjusted optical biometry [1], and combined applanation vector-A/B-scan biometry [8]. However, previous studies have inadequately evaluated preoperative AL measurements, creating a significant gap in accurately determining IOL power for macula-off RRD, and there is no consensus on the optimal method of AL measurement for IOL calculation in such groups of patients. In addition, these research studies contained some limitations: (1) lack of detailed preoperative parafoveal detachment and confirmatory imaging for postoperative foveal reattachment; (2) calculation of AL errors using mean errors instead of absolute errors, which did not refer to the true errors; and (3) the use of complicated methods of AL estimation.

In this study, we created two novel algorithms for AL selection for IOL power calculation in patients with macula-off RRD. The results of mean absolute prediction errors (MAE) of preoperative AL guided by the algorithms were analyzed and compared with those of affected eyes using IOL Master 700 and A-scan, together with those of fellow eyes using IOL Master 700. Complete parafoveal detachment was confirmed preoperatively by fundus examination or optical coherence tomography (OCT), and postoperative foveal reattachment was confirmed by OCT in every patient. In addition, a literature review of studies focusing on preoperative AL estimation in patients with macula-off RRD was conducted.

## Patients and methods

This was designed as a retrospective descriptive study of prospectively collected patient data in a tertiary-level hospital. From January 2018 to December 2023, patients undergoing combined phacovitrectomy for macula-off RRD at the Ophthalmology Department, Rajavithi Hospital, Bangkok, Thailand, gave written informed consent and were enrolled in this study, which followed the tenets of the Declaration of Helsinki and was approved by the Ethics Committee of Rajavithi Hospital (approval No.187/2563). All the patients in the study were diagnosed with RRD with grade 5 foveal detachment (complete parafoveal detachment), as previously described by Klaas et al. [9] All cases had phacoemulsification with in-the-bag IOLs and vitrectomy with tamponade agents, either C_3_F_8_ or silicone oil. We excluded RRD patients who had the following conditions: (1) combined choroidal detachment in any area; (2) missing preoperative or postoperative report of AL or A-scan measurement; (3) being treated with the scleral buckling procedure (SBP); (4) retaining silicone oil tamponade without removal; (5) having had failed RRD surgery; and (6) any other ocular problems affecting biometric measurements, such as opaque corneal and lens dislocation.

Data collected encompassed patient demographics, preoperative and postoperative ocular biometry, type of IOL used, operative details including intraoperative tamponade, postoperative dilated fundus examination and OCT results. Spectral domain OCT (Spectralis OCT, Heidelberg, Germany) was performed for all patients to confirm postoperative foveal reattachment.

The instruments for ocular biometry performed in the preoperative and postoperative stages were IOL Master 700 (Zeiss, Germany), and an A-scan by VuMAX (Sonomed, USA). IOL power was calculated using the manufacturer’s recommended A-constant, and the SRK/T formula because of its accuracy across a range of AL [10]. The AL of the affected eye, a key focus of this study, was assessed and estimated using five approaches, including two newly developed algorithms. Preoperative AL measurement was conducted by the main three methods as follows: (1) IOL Master for affected eye (AF-OpB); (2) IOL Master for fellow eye (FE-OpB); and (3) A-scan for affected eye (AF-A scan). In addition, two novel algorithms for selecting preoperative AL for IOL power calculation were utilized. These included a simple algorithm (S-Algor) and advanced algorithm (A-Algor). AL was measured by experienced operators. When using the optical biometry, only measurements with a signal-to-noise ratio above 2 were selected. Ultrasound measurements were obtained using the immersion technique with the patient in the supine position. Ten reliable AL readings were taken for all patients, and the mean value was used for subsequent calculations.

### The formation of two algorithms

Two novel algorithms included S-Algor and A-Algor. S-Algor was based on selecting the higher value of AL between AF-OpB and FE-OpB. However, if the AF-IOL could not be obtained, the FE-OpB was used. A-Algor was multi-tiered and constructed based on statistically analyzed population data from 200 healthy individuals to mitigate possible errors from preoperative anisometropia.

**Fig. 1:**
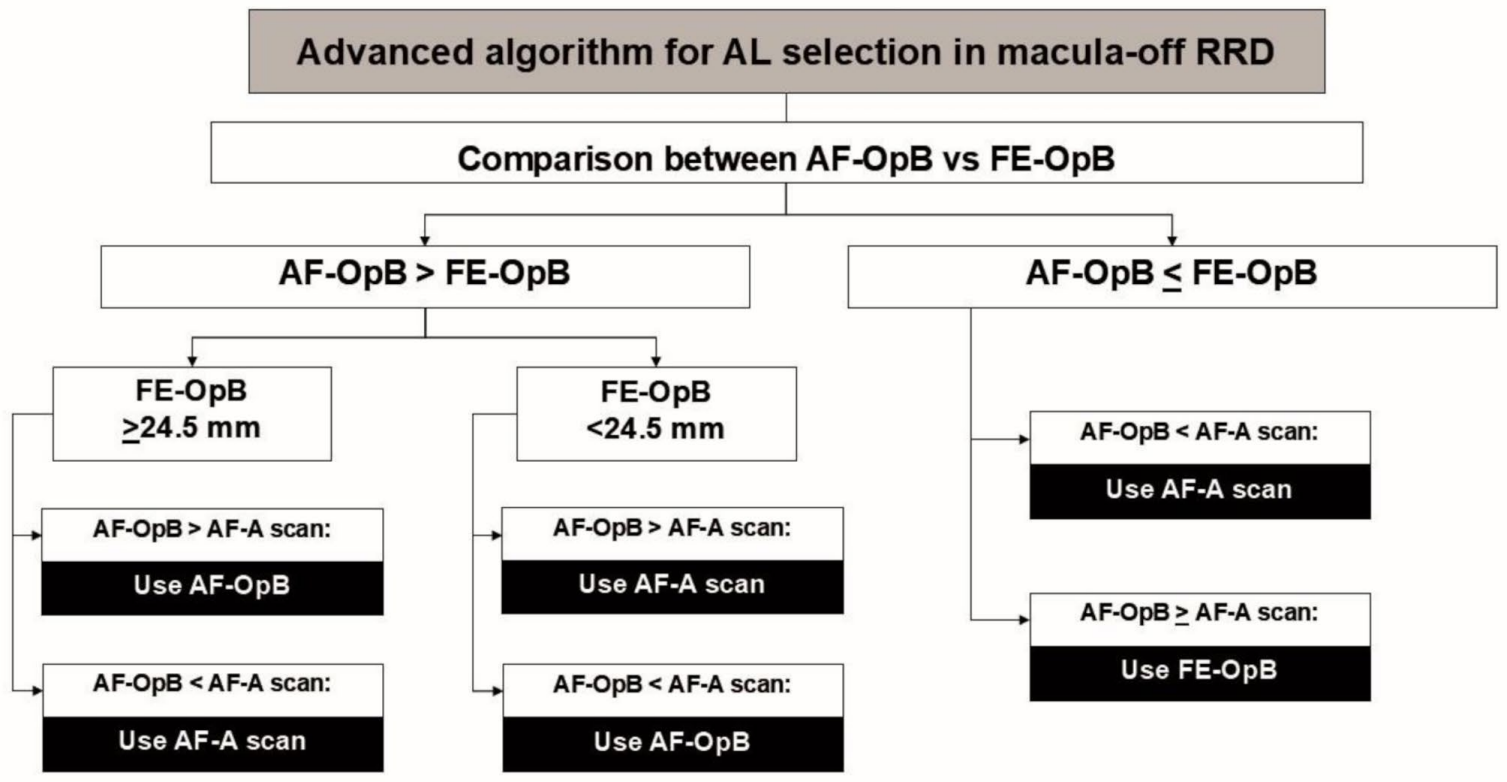
Advanced algorithm for axial selection in macula-off rhegmatogenous retinal detachment (AL = axial length, AF-OpB = affected eye’s IOL Master optical biometry, FE-OpB = fellow eye’s IOL Master optical biometry, AF-A scan = affected eye’s A-scan biometry)

The creation of A-Algor (Fig. 1) was based on a few key observations. Firstly, assuming isometropia in all cases, the AL of the affected eye measured by the IOL Master 700 tends to be the erroneously shortest according to ocular biometry across the three methods of AL measurement (AF-OpB, FE-OpB and AF-A scan) [4, 6, 11]. Secondly, an increase in AL corresponds to greater asymmetry between eyes [12, 13]. Thirdly, employing A-scan for calculating IOL power in macula-off RRD is less likely to induce a myopic shift compared to using optical biometry for IOL calculation in such cases [7, 11, 14]. Regarding a population-based dataset obtained from optical biometry of our 200 healthy cases, the assumption of normal distribution was proven using the Kolmogorov Smirnov test. The longer eye was chosen from each participant for descriptive analysis of the data. Mean AL was 23.8 *±* 1.2 (range, 21.6–28.5) mm. The cut-off point of AL to indicate significant interocular asymmetries in AL was set at 24.5 mm as previously reported [13]. This value was applicable with our population data since it lay within one standard deviation of the mean. In addition, with linear regression analysis of our data, this cut-off point was related to AL’s interocular difference at 0.5 mm which was reasonable and acceptable. Therefore, the cut-off point of 24.5 mm for FE-OpB was applied if the AF-OpB was longer than the FE-OpB, indicating significant interocular asymmetries in AL. (Fig. 1, left arm) Preoperatively, FE-OpB equal to or longer than 24.5 mm indicates a tendency for a longer AL in the status of reattached retina of an affected eye. FE-OpB is disregarded, with only AF-OpB and AF-A scan considered for the final decision. The choice between AF-OpB and AF-A scan is made based on the longer AL. The same procedure is followed for FE-OpB shorter than 24.5 mm, with the shorter AL of the affected eye selected from the comparison between AF-OpB and AF-A scans.

For the right arm of Fig. 1, in view of the fact that AF-OpB represents the possibility of erroneously shortest AL among three different methods of AL estimations, it is disregarded for the final decision. Here, AF-A scan plays a crucial role in determining the ultimate choice. However, if the AF-OpB is equal or longer than the AF-A scan, contradicting the observed fact, the FE-OpB should be selected.

### Surgical technique and outcome measures

All patients underwent routine baseline preoperative clinical evaluation and preparation [15, 16]. All the surgical procedures were performed by a single surgeon (S.S.). Phacoemulsification was performed through a 2.75-mm clear cornea incision at the superotemporal quadrant of the right eye or superonasal quadrant of the left eye. A Sensar AR40e IOL (Abbot Medical Optics, CA, USA) was injected into the capsular bag in all cases. Three-port 23-gauge pars plana vitrectomy was performed and sub-retinal fluid was internally aspirated without the assistance of heavy liquid, after which fluid-air exchange was rendered, followed by retinopexy using endophotocoagulation. Intraocular tamponade was achieved using either gas (C_3_F_8_) or silicone oil.

After the foveal reattachment was confirmed by spectral domain OCT, AL was measured using IOL Master 700 at 8–12 weeks after the surgery or silicone oil removal. In summary, there were five AL options to choose from for preoperative IOL calculation: AF-OpB, FE-OpB, AF-A scan, S-Algor’s AL, and A-Algor’s AL. These preoperative estimations were compared with the postoperative AL of the affected eye. The primary outcome measure was the average difference between the postoperative AL of the affected eye and the values measured/estimated by the five methods. The MAE was selected as the main outcome instead of the mean prediction error (ME), as ME can misrepresent the error by averaging positive and negative values [6].

### Statistical analysis

The minimum sample size required for estimating correlations above 0.70 at an alpha level of 0.05 (corresponding Z_α/2_ = 1.96) and 80% power (corresponding Z_β_ = 0.842) was 14 patients. Statistical analysis was performed using the IBM SPSS Statistics for Windows, Version 20.0 (Armonk, NY: IBM Corp; 2011).

The Kolmogorov–Smirnov test was employed to assess the normality of data distribution. Kruskal-Wallis H test was used to compare different medians, while Wilcoxon signed rank test was used to evaluate the differences between preoperative and postoperative measurements within the same individual. Bland–Altman analysis was performed with bootstrapping to estimate bias and upper and lower limits of agreement (LoA) between preoperative and postoperative measurements. A *P*-value of < 0.05 was considered statistically significant.

## Results

A total of 56 patients (56 eyes) were included in the study. Mean age was 56 *±* 11 (range, 28–78) years old, and 55% (31/56) of participants were female. Right eye was included for 61% (34/56), and 61% (34/56) of cases had grade B or less proliferative retinopathy. Perfluoropropane gas was used as a tamponade agent in 93% (52/56) and silicone oil was used for tamponade agent for the other 7% (4/56). Regarding preoperative biometry data performed by IOL Master, AL of the affected eyes was achievable in 51 eyes and the median AF-OpB was 23.25 mm. (Table 1) All cases obtained FE-OpB. Median FE-OpB and AF-A scan values were 23.82 mm and 23.33 mm respectively, while median postoperative AL of the affected eye was 23.68 mm. The median AL obtained from S-Algor and A-Algor were 23.82 mm and 23.53 mm respectively.

**Table 1:** Preoperative and postoperative axial length of the included eyes measured/estimated by five different methods (*n* = 56)

Methods	*N* (eyes)	Median AL (mm)	*P* value*
Preoperative			
AF-OpB	51	23.25 (14.07–29.24)	0.024
FE-OpB	56	23.82 (22.13–30.25)	0.175
AF-A Scan	56	23.33 (21.31–30.73)	0.025
S-Algor	56	23.82 (22.14–30.25)	0.000
A-Algor	56	23.53 (21.99–30.73)	0.191
Postoperative			
AL of the affected eye measured by IOL Master	56	23.68 (22.33–28.89)	-

AL = axial length, AF-OpB = affected eye’s IOL Master optical biometry, FE-OpB = fellow eye’s IOL Master optical biometry, AF-A scan = affected eye’s A-scan biometry, S-Algor = simple algorithm method, A-Algor = advanced algorithm method

*Wilcoxon signed rank test

Wilcoxon signed rank test was used to compare preoperative and postoperative AL measurements of the affected eye. There were statistically significant differences between the preoperative AL measurements (AF-OpB, AF-A scan, and S-Algor) and the postoperative AL of the affected eye (Table 1).

**Fig. 2:**
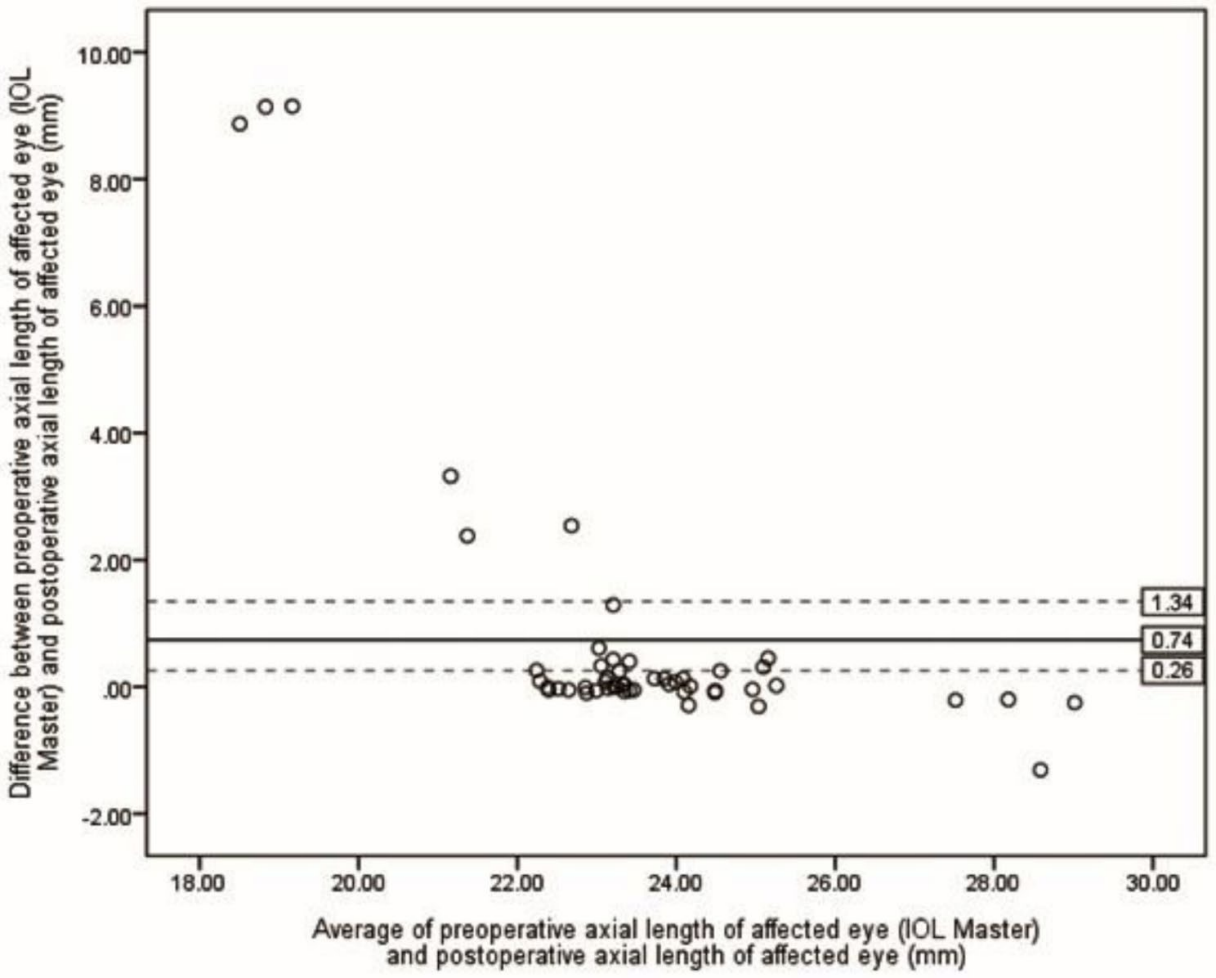
Bland-Altman plot indicating differences between preoperative axial length of affected eye measured by IOL Master and postoperative axial length of affected eye as a function of averages

**Fig. 3:**
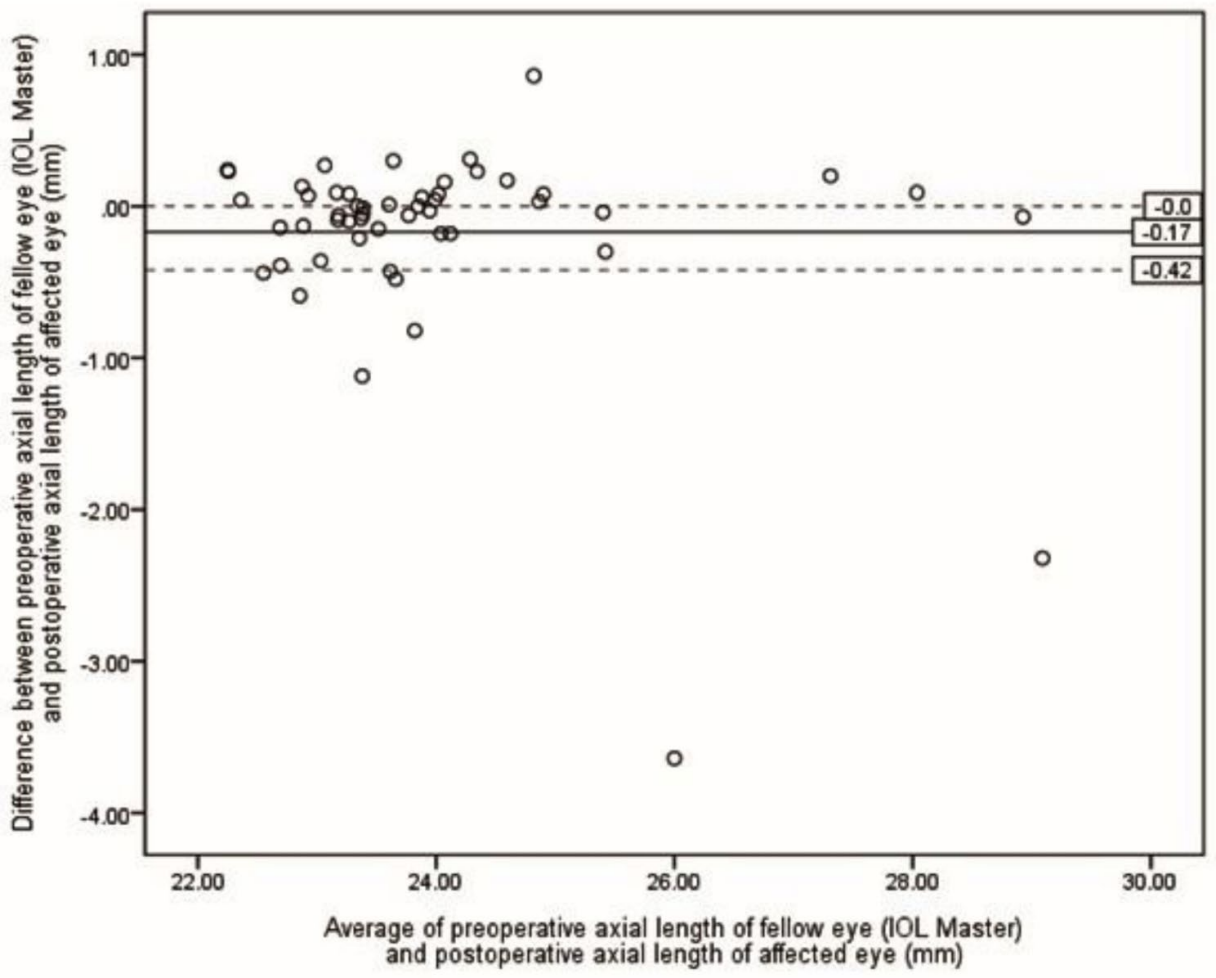
Bland-Altman plot indicating differences between preoperative axial length of fellow eye measured by IOL Master and postoperative axial length of affected eye as a function of averages

**Fig. 4:**
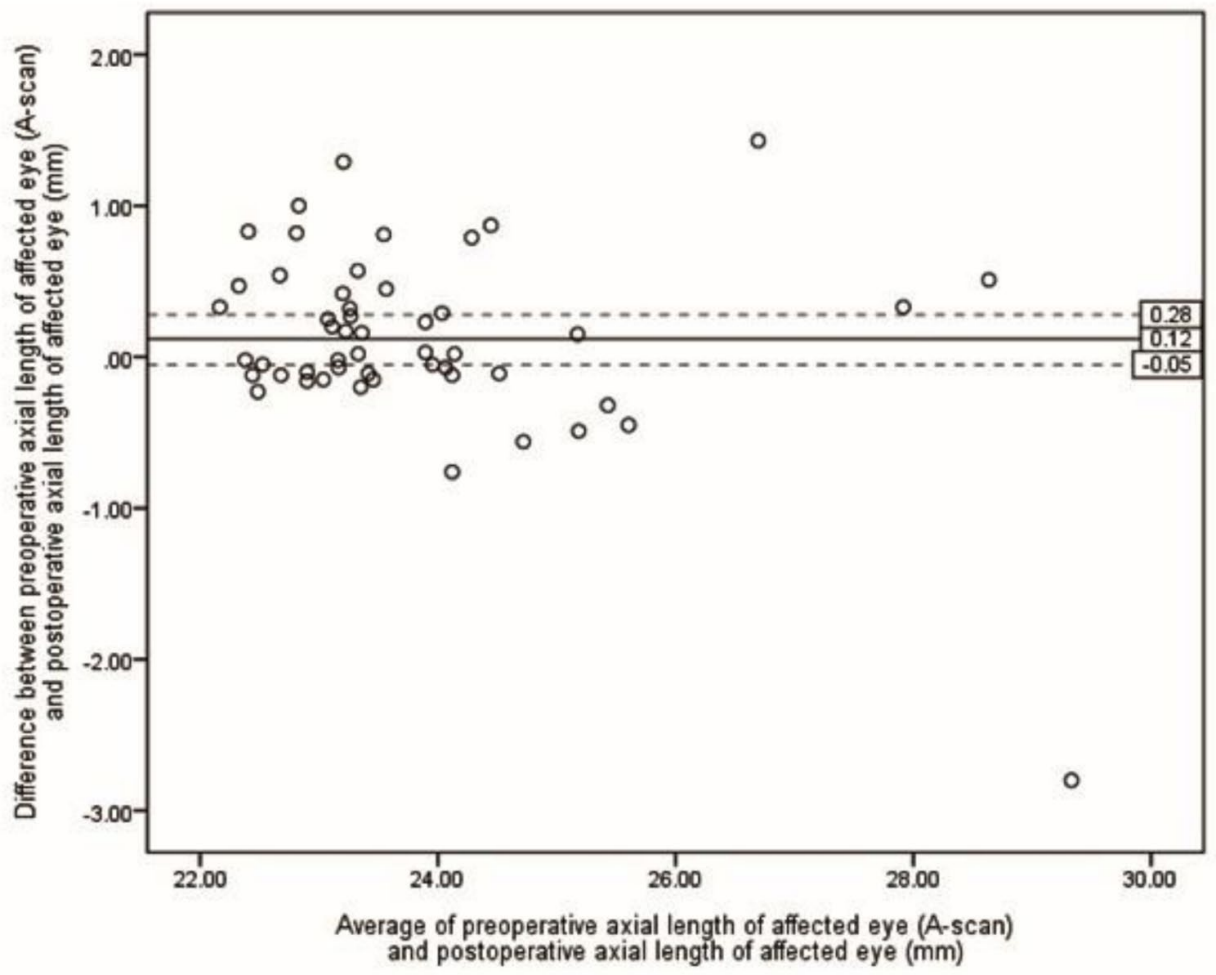
Bland-Altman plot indicating differences between preoperative axial length of affected eye measured by A-scan and postoperative axial length of affected eye as a function of averages

**Fig. 5:**
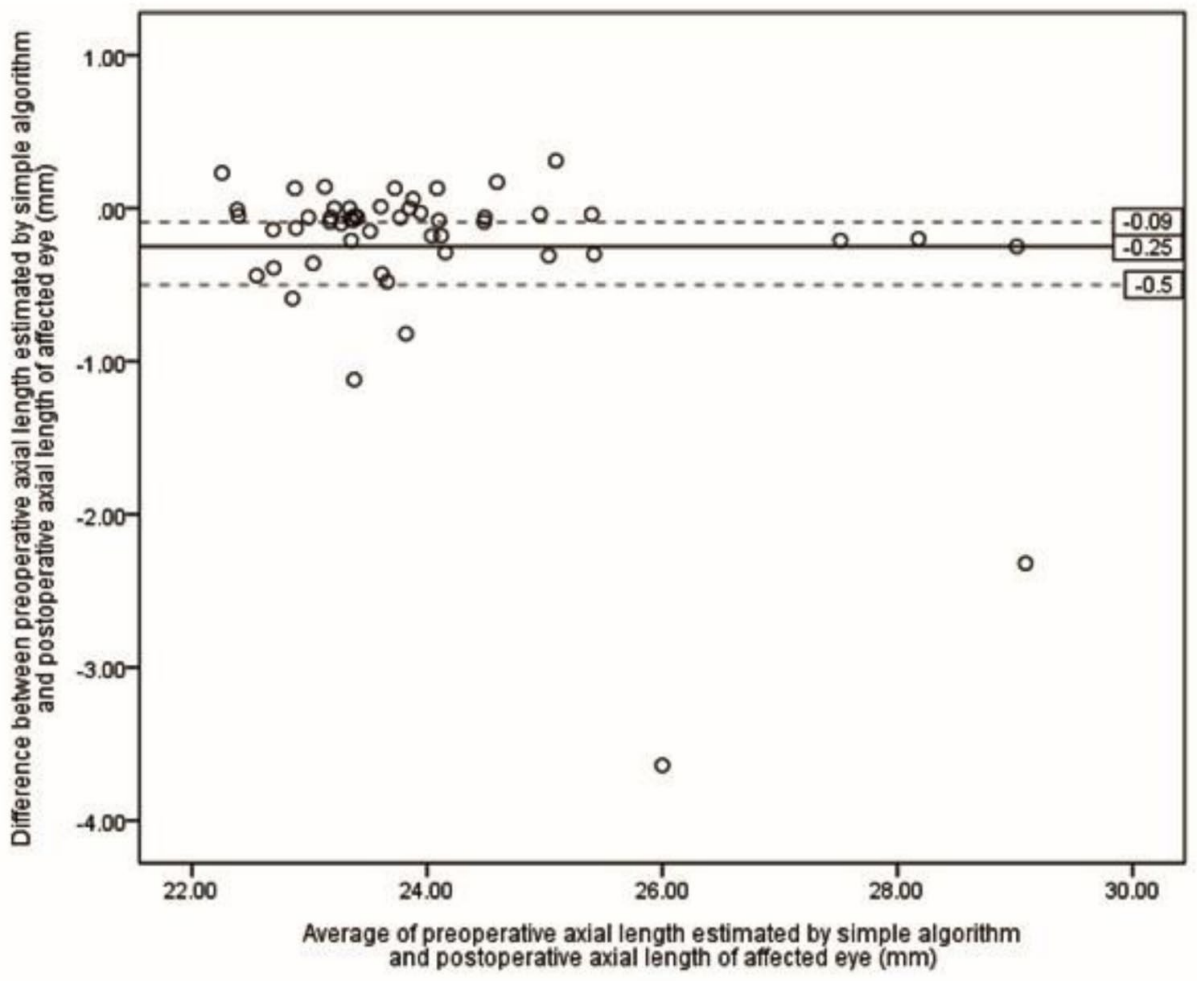
Bland-Altman plot indicating differences between preoperative axial length of affected eye estimated by simple algorithm and postoperative axial length of affected eye as a function of averages

**Fig. 6:**
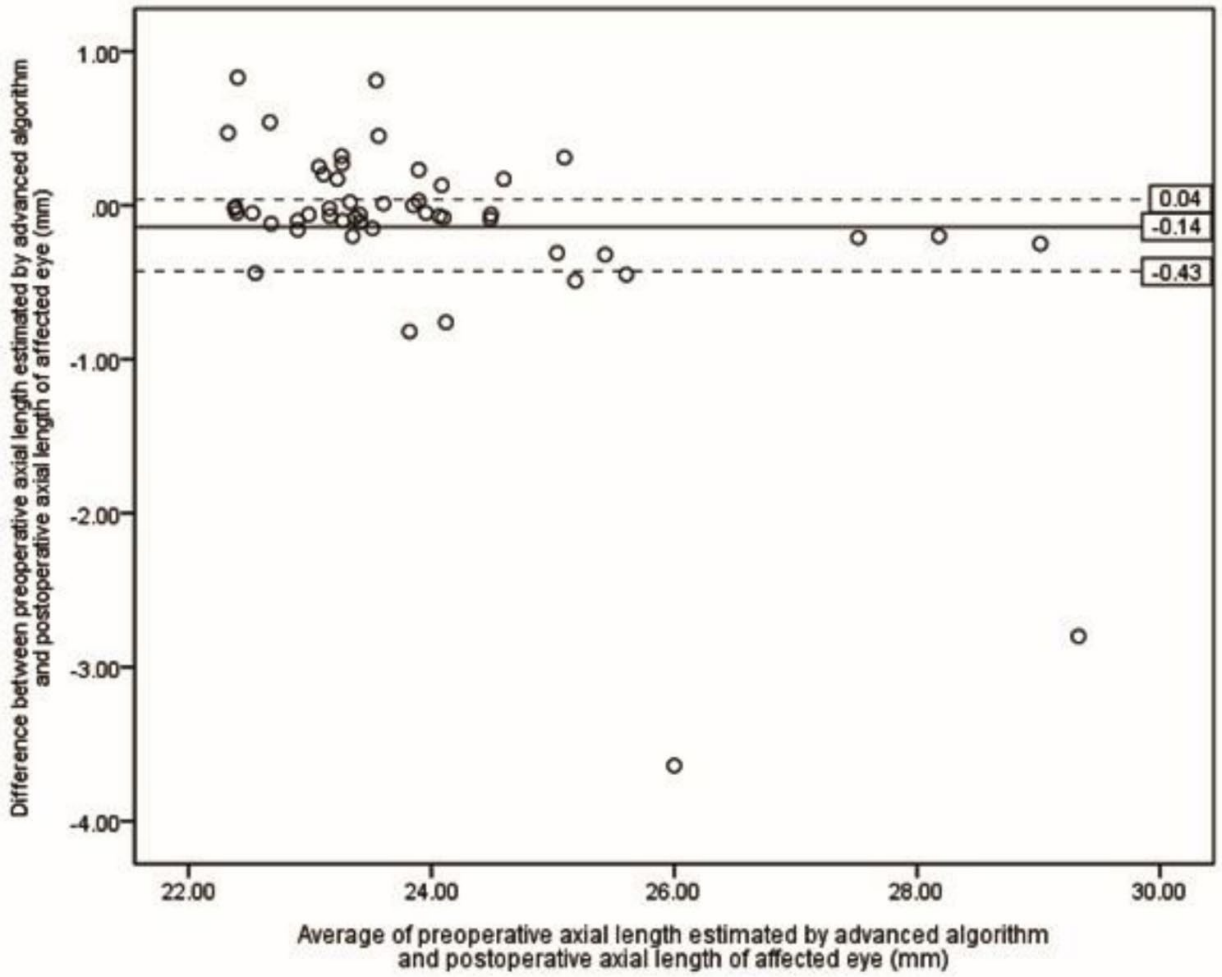
Bland-Altman plot indicating differences between preoperative axial length of affected eye estimated by advanced algorithm and postoperative axial length of affected eye as a function of averages

Table 2 presents the ME and MAE of differences between preoperative selections of ALs and postoperative ALs. The lowest MAE was achieved by the S-Algor (0.31 *±* 0.55 mm), followed by FE-OpB (0.34 *±* 0.60 mm) and A-Algor (0.36 *±* 0.62 mm). The Kruskal-Wallis H test showed that there was no statistical difference between MAE resulting from all five methods (*P* = 0.118). However, when the five eyes for which we were unable to obtain preoperative AF-OpB were removed in each method, the MAE values were reduced in all groups (Table 3), and the Kruskal-Wallis H test revealed that there was no statistical difference between the means of the measured ALs (*P* = 0.081). Figures 2, 3, 4, 5 and 6 show the Bland-Altman plots of different preoperative selections of AL and postoperative AL measured by the IOL Master. The Bland-Altman plot demonstrated good agreement between the preoperative AL measurements (using FE-OpB, S-Algor, and A-Algor) and the postoperative AL measurements obtained using the IOL Master.

**Table 2:** Mean prediction errors and mean absolute prediction errors of differences between preoperative Estimation of axial length and postoperative axial length

	AF-OpB (*n* = 51)	FE-OpB (*n* = 56)	AF-A scan (*n* = 56)	S-Algor (*n* = 56)	A-Algor (*n* = 56)
Mean prediction errors (mm)	0.73 ± 2.22 (-1.31 to 9.15)	-0.16 ± 0.67 (-3.64 to 1.30)	0.20 ± 0.81 (-2.80 to 4.27)	-0.23 ± 0.64 (-3.64 to 1.30)	-0.13 ± 0.71 (-3.64 to 1.30)
Mean absolute prediction errors (mm)	0.87 ± 2.17 (0 to 9.15)	0.34 ± 0.60 (0 to 3.64)	0.47 ± 0.69 (0.02 to 4.27)	0.31 ± 0.55 (0 to 3.64)	0.36 ± 0.62 (0 to 3.64)

AF-OpB = affected eye’s IOL Master optical biometry, FE-OpB = fellow eye’s IOL Master optical biometry, AF-A scan = affected eye’s A-scan biometry, S-Algor = simple algorithm method, A-Algor = advanced algorithm method

**Table 3:** Mean prediction errors and mean absolute prediction errors of differences between preoperative Estimation of axial length and postoperative axial length (the five eyes for which we were unable to obtain preoperative AF-OpB were removed)

	AF-OpB (*n* = 51)	FE-OpB (*n* = 51)	AF-A scan (*n* = 51)	S-Algor (*n* = 51)	A-Algor (*n* = 51)
Mean prediction errors (mm)	0.74 ± 2.22 (-1.31 to 9.15)	-0.17 ± 0.66 (-3.64 to 0.86)	0.12 ± 0.62 (-2.80 to 1.43)	-0.25 ± 0.62 (-3.64 to 0.31)	-0.14 ± 0.71 (-3.64 to 0.83)
Mean absolute prediction errors (mm)	0.87 ± 2.17 (0-9.15)	0.32 ± 0.60 (0 to 3.64)	0.41 ± 0.48 (0.02 to 2.80)	0.28 ± 0.55 (0 to 3.64)	0.35 ± 0.63 (0 to 3.64)

AF-OpB = affected eye’s IOL Master optical biometry, FE-OpB = fellow eye’s IOL Master optical biometry, AF-A scan = affected eye’s A-scan biometry, S-Algor = simple algorithm method, A-Algor = advanced algorithm method

## Discussion

The main factors that affect IOL power calculations are AL and corneal power. However, in eyes with macula-off RRD, the AL is the only key variable factor for IOL power determination. Previous studies sought ways to estimate preoperative AL in such groups of patients; however, instead of identifying AL errors between preoperative and postoperative measurements, some authors focused on postoperative refractive outcome, which could be crucially influenced by corneal power [4–7]. To completely evaluate the AL in preoperative macula-off RRD and in postoperative retina reattachment, a number of conditions should be met: (1) detailed para/perifoveal detachment [9]; (2) consistent use of IOL formula; (3) confirmatory foveal reattachment with OCT imaging; and (4) the use of the immersion technique for A-scan instead of the contact method. Our literature review identified a few studies which have explored the different biometry techniques in estimating preoperative AL in IOL power calculation for combined phacovitrectomy in macula-off RRD. (Table 4) These included optical biometry and A-scan for same and fellow eyes [4, 14, 17, 18], user-adjusted optical biometry [1], and combined applanation vector-A/B-scan biometry [8, 17].

**Table 4:** Literature review of studies for preoperative axial length Estimation in eyes with macula-off retinal detachment

Authors, year	Eyes with macula-off	Detailed foveal detachment	IOL formula	Operations	Tamponade agent	Confirmatory foveal reattachment with OCT imaging	Postoperative axial length comparison	Methods of preoperative axial length measurements/estimations						Evaluation of errors in axial length estimation	Recommended methods for selecting preoperative axial length (Best outcome)
								Ocular biometry in affected eye	Ocular biometry in fellow eye	Ultrasound A-scan in affected eye	User-adjusted axial length	Vector-A/B-scan ultrasound	Algorithm		
Current work, 2025	56	Yes	SRK/T	Phacovitrectomy surgery (56)	Gas (52), silicone oil (4)	Yes	Yes (56)	IOL Master 700	IOL Master 700	Immersion	-	-	Yes	Mean absolute errors	Simple algorithm
Helaly et al. [17], 2024	100	NA	NA	Phacovitrectomy surgery (100)	NA	No	Yes (100)	IOL Master 700 and ARGOS	IOL Master 700 and ARGOS	Contact	ARGOS	Yes	-	Mean errors	Vector-A/B-scan ultrasound
Kimura et al. [14], 2023	42	NA	SRK/T	Phacovitrectomy surgery (33)	Gas	No	Yes (42)	OA-2000	OA-2000	Contact	-	-	-	Mean absolute errors	Fellow-eye biometry and A-scan of affected eye
Liu et al. [18], 2022	31	NA	SRK/T	Vitrectomy alone (22), Phacovitrectomy surgery (9)	Silicone oil	Yes	Yes (31)	-	IOL Master 700	Contact	-	-	-	Mean errors	Fellow-eye optical biometry
El-Khayat et al. [6], 2019	42	NA	SRK/T, Haigis, Hoffer-Q	Phacovitrectomy surgery (42)	PFCL, Gas, silicone oil	No	No	IOL Master (unknown version)	-	Contact	-	-	-	No	Fellow-eye biometry (based on refractive outcome)
Pak et al. [7], 2019	33	Yes	SRK/T	Phacovitrectomy surgery (33)	Gas	No	No	IOL Master (unknown version), contact A-scan	IOL Master (unknown version), contact A-scan	Contact	-	-	-	No	A-scan when it was similar to its fellow eye (based on refractive outcome)
Pongsachareonnont et al. [11], 2018	16	NA	NA	Phacovitrectomy surgery (16)	Gas	No	Yes (16)	IOL Master 500	-	Immersion	-	-	-	Mean errors	Both optical biometry and ultrasound
Abou-Shousha et al. [8], 2016	100	NA	NA	Phacovitrectomy surgery (42)	Gas, silicone oil	No	Yes (100)	IOLMaster (unknown version)	-	Contact	-	Yes	-	Mean errors	Vector-A/B-scan ultrasound (based on refractive outcome)
Rahman et al. [1], 2016	22	NA	NA	Phacovitrectomy surgery (22)	NA	No	Yes (13)	IOL Master Version 5.4	IOL Master Version 5.4	Contact	IOL Master Version 5.4	-	-	Mean errors	User-adjusted method -Suggest fellow eye’s optical biometry
Kim et al. [5], 2015	26	NA	SRK/T	Phacovitrectomy surgery (26)	Gas	No	Yes (20)	IOL Master (unknown version)	-	Immersion	-	-	-	No	Fellow-eye biometry (based on refractive outcome)
Rahman et al. [4], 2014	54	NA	SRK/T	Phacovitrectomy surgery (54)	Gas (47), silicone oil (7)	No	Yes (40)	IOL Master Version 5.4	IOL Master Version 5.4	Contact (some cases)	-	-	-	No	Both optical biometry and ultrasound (based on refractive outcome)

OCT = optical coherence tomography, NA = not available, PFCL = perfluorocarbon liquid

Rahman et al. evaluated the accuracy of user-adjusted AL measured by optical biometry [1]. With this method, AL measurements were manually adjusted by a skilled biometry operator by shifting the signal peak default to a more posterior peak. AL obtained from this method was not statistically significantly different from the postoperative optical biometry. However, in addition to the complexity of the method, the result of posterior multiple peaks in the scans can cause a problem, as this small study did not clearly demonstrate whether the affected eye’s A-scan or the fellow eye’s IOL Master was used in cases of images with no defined single posterior peak.

Combined applanation vector-A/B-scan biometry was based on A-scan measurement supplemented by contact B-scan ultrasonography [8, 17]. Although the authors concluded that the vector-A/B-scan offered the better model of the actual AL measurement in the patients compared with optical biometry and A-scan ultrasound, the method encompassed some limitations, including a complexity level necessitating a skillful operator, and the tendency of corneal compression by contact A-scan ultrasonography. A recent study from the same authors evaluated preoperative AL in 100 eyes using vector-A/B-scan biometry and ARGOS swept-source optical coherence biometer [17]. Enhanced Retinal Visualization (ERV) mode in ARGOS enables precise localization by allowing manual cursor alignment with the highest spike, ensuring accuracy in cases such as detached macula. Although the study approved the value of ERV mode for ARGOS in making every eye measurable, and selected vector-A/B-scan ultrasound as the most accurate method for measuring AL in same eye, the study included the following limitations: (1) no detailed use of IOL formula; (2) lack of confirmatory imaging for foveal reattachment; and (3) the use of ME, instead of MAE, to demonstrate the accuracy of the methods, which may lead to false interpretation of the errors. Our group sought an effective, manipulation-free method to estimate preoperative AL for IOL power calculation in macula-off RRD patients. As such, we utilized automatic measurement for AL without user adjustment. MAE was also used as the primary outcome to demonstrate the accuracy of the methods.

Among the studies lacking focus on AL evaluation using MAE, Kimura et al. reported the MAE between preoperative AL (AF-OpB, FE-OpB, and AF-A scan) and postoperative measurements of AL in an affected eye, which were 1.22 ± 2.40 mm, 0.35 ± 0.49 mm, and 0.24 ± 0.24 mm respectively [14]. They concluded that FE-OpB or AF-A scan was more accurate than AF-OpB in cases with macular detachment. Although a different optical biometer (OA-2000) was used in the study, the results of MAE are consistent with those of our work. Some limitations in Kimura’s study included performance of A-Scan using the contact technique, and its lack of confirmatory foveal reattachment by OCT imaging.

Our work utilized the decision algorithm based on the AL obtained from the routine ocular biometry including IOL Master and A-scan using the immersion technique. While S-Algor can be simply followed by selecting the higher AL between AF-OpB vs. FE-OpB for IOL power calculation, A-Algor is based on statistically analyzed population data from 200 healthy individuals in order to mitigate possible errors from preoperative anisometropia. The selection of preoperative AL using A-Algor (MAE, 0.35 ± 0.63 mm) achieved lower MAE than AF-OpB and AF-A scan, while S-Algor resulted in the lowest MAE of 0.31 ± 0.55 mm compared with the other four methods. The MAE of S-Algor and other methods were even lower (0.28 ± 0.55 mm) after removing five eyes for which we were unable to obtain preoperative AF-OpB. Regarding attempts for preoperative AL estimation, some authors have tried to identify the correlation between the height of RRD and the error in AL measurement; [5, 14] however, the height of RRD is changeable and is not measurable in all cases. Besides the detached macula, the significant preoperative anisometropia remained as a major challenge in preoperative AL estimation, which may have caused the outliers in our results [3].

Some limitations of this study should be noted. First, its retrospective nature renders it at risk of bias. Second, the small number of patients could carry a potential risk of patient selection bias. Third, the inclusion of anisometropia caused higher errors and numbers of outliers. In contrast, the strengths of our study included the prospectively collected data, which addressed the limitations which caused biases in previous studies. In addition, AL as a key factor for IOL power calculation in macula-off RRD was mainly evaluated and estimated using modern and standard ocular biometry, as well as newly created simple and advanced algorithms.

In conclusion, for patients with macula-off RRD, we favour the use of a simple algorithm—selecting the higher AL between AF-OpB and FE-OpB—for preoperative AL in IOL power calculation. However, if AF-OpB cannot be obtained preoperatively, phacovitrectomy using FE-OpB could serve as a reliable alternative.

## Data Availability

No datasets were generated or analysed during the current study.

## Abbreviations

RRD: Rhegmatogenous retinal detachment
IOL: Intraocular lens
MAE: Mean absolute prediction errors
OCT: Optical coherence tomography
SBP: Scleral buckling procedure
AF-OpB: IOL Master for affected eye
FE-OpB: IOL Master for fellow eye
AF-A scan: A-scan for affected eye
S-Algor: Simple algorithm
A-Algor: Advanced algorithm
LoA: Limits of agreement
ERV: Enhanced Retinal Visualization

## References

[1] Rahman R, Kolb S, Bong CX, Stephenson J. Accuracy of user-adjusted axial length measurements with optical biometry in eyes having combined phacovitrectomy for macular-off rhegmatogenous retinal detachment. J Cataract Refract Surg. 2016;42(7):1009–14. 27492099 10.1016/j.jcrs.2016.04.030

[2] Daud F, Daud K, Popovic MM, Yeung S, You Y, Cruz Pimentel M, et al. Combined versus sequential Pars plana vitrectomy and phacoemulsification for macular hole and epiretinal membrane: A systematic review and Meta-Analysis. Ophthalmol Retina. 2023;7(8):721–31. 37030392 10.1016/j.oret.2023.03.017

[3] Olsen T. Calculation of intraocular lens power: a review. Acta Ophthalmol Scand. 2007;85(5):472–85. 17403024 10.1111/j.1600-0420.2007.00879.x

[4] Rahman R, Bong CX, Stephenson J. Accuracy of intraocular lens power Estimation in eyes having phacovitrectomy for rhegmatogenous retinal detachment. Retina. 2014;34(7):1415–20. 24384617 10.1097/IAE.0000000000000072

[5] Kim YK, Woo SJ, Hyon JY, Ahn J, Park KH. Refractive outcomes of combined phacovitrectomy and delayed cataract surgery in retinal detachment. Can J Ophthalmol. 2015;50(5):360–6. 26455971 10.1016/j.jcjo.2015.07.003

[6] El-Khayat AR, Brent AJ, Peart SAM, Chaudhuri PR. Accuracy of intraocular lens calculations based on fellow-eye biometry for phacovitrectomy for macula-off rhegmatogenous retinal detachments. Eye (Lond). 2019;33(11):1756–61. 31182834 10.1038/s41433-019-0485-0 PMC7002476

[7] Pak KY, Park KH, Park SW, Byon IS, Lee JE. Comparison between refractive outcomes between macula-on and macula-off retinal detachments after phaco-vitrectomy. Jpn J Ophthalmol. 2019;63(4):310–6. 31006060 10.1007/s10384-019-00667-6

[8] Abou-Shousha M, Helaly HA, Osman IM. The accuracy of axial length measurements in cases of macula-off retinal detachment. Can J Ophthalmol. 2016;51(2):108–12. 27085268 10.1016/j.jcjo.2015.12.011

[9] Klaas JE, Siedlecki J, Steel DH, Laidlaw DAH, Priglinger S. How should we report the foveal status in eyes with macula-off retinal detachment? Eye (Lond). 2023;37(2):228–34. 35505112 10.1038/s41433-022-02074-7 PMC9873750

[10] Retzlaff JA, Sanders DR, Kraff MC. Development of the SRK/T intraocular lens implant power calculation formula. J Cataract Refract Surg. 1990;16(3):333–40. 2355321 10.1016/s0886-3350(13)80705-5

[11] Pongsachareonnont P, Tangjanyatam S. Accuracy of axial length measurements obtained by optical biometry and acoustic biometry in rhegmatogenous retinal detachment: a prospective study. Clin Ophthalmol. 2018;12:973–80. 29872256 10.2147/OPTH.S165875 PMC5973443

[12] Rajan MS, Bunce C, Tuft S. Interocular axial length difference and age-related cataract. J Cataract Refract Surg. 2008;34(1):76–9. 18165085 10.1016/j.jcrs.2007.08.023

[13] Albarran-Diego C, Poyales F, Lopez-Artero E, Garzon N, Garcia-Montero M. Interocular biometric parameters comparison measured with swept-source technology. Int Ophthalmol. 2022;42(1):239–51. 34417946 10.1007/s10792-021-02020-8 PMC8803707

[14] Kimura S, Hosokawa MM, Shiode Y, Matoba R, Kanzaki Y, Goto Y, et al. Accuracy of ultrasound vs. Fourier-domain optic biometry for measuring preoperative axial length in cases of rhegmatogenous retinal detachment. Jpn J Ophthalmol. 2023;67(6):645–51. 37561309 10.1007/s10384-023-01018-2

[15] Silpa-Archa S, Kumsiang K, Preble JM. Endophthalmitis after Pars plana vitrectomy with reused single-use devices: a 13-year retrospective study. Int J Retina Vitreous. 2021;7(1):2. 33407931 10.1186/s40942-020-00274-5 PMC7788751

[16] Silpa-Archa S, Papirachnart A, Singhanetr P, Preble JM. Risk factors for endophthalmitis after cataract surgery in diabetic patients: a case control study. Int J Ophthalmol. 2019;12(3):417–23. 30918810 10.18240/ijo.2019.03.11 PMC6423383

[17] Helaly HA, Elnaggar OR, Abou Shousha M, Elhady AM. Accuracy of axial length measurements by two Swept-Source optical coherence tomography biometers in Macula-off rhegmatogenous retinal detachment eyes. Clin Ophthalmol. 2024;18:3321–34. 39582494 10.2147/OPTH.S469094 PMC11586123

[18] Liu R, Li H, Li Q. Differences in axial length and IOL power based on alternative A-Scan or Fellow-Eye biometry in Macula-Off rhegmatogenous retinal detachment eyes. Ophthalmol Ther. 2022;11(1):347–54. 34878642 10.1007/s40123-021-00439-x PMC8770769

